# Twin pregnancy with incomplete hydatidiform mole and coexistent normal live fetus: A case report and review of the literature

**DOI:** 10.1097/MD.0000000000035072

**Published:** 2023-11-03

**Authors:** Carlos Ernesto Mora-Palazuelos, Luis Garay-Vizcarra, Paul Gonzalez-Quintero, Daniel Miranda-Rosas, Eri Peña-Martínez, Erik Rene Lizarraga-Verdugo, Saul Armando Beltrán-Ontiveros, Jose Geovanni Romero-Quintana

**Affiliations:** a Center for Research and Teaching in Health Sciences, Autonomous University of Sinaloa, Culiacan, Sinaloa, Mexico; b Women’s Hospital of Sinaloa, Ministry of Health, Culiacan, Sinaloa, Mexico; c Sinaloa Pediatric Hospital, Ministry of Health, Culiacan, Sinaloa, Mexico; d Faculty of Biological and Chemical Sciences Autonomous University of Sinaloa, Culiacan, Sinaloa, Mexico.

**Keywords:** case report, immunohistochemistry, incomplete mole, normal live fetus, twin pregnancy

## Abstract

**Rationale:**

A pregnancy with incomplete mole is very rare case. Hydatidiform mole (HM) with live fetus is associated with a risk of a wide variety to maternal and fetal complications. The incidence of a normal live fetus and an incomplete mole such as the case we describe is extremely rare.

**Patient concern:**

We report a case of multiparous 34-year-old at Culiacan Mexico woman with incomplete mole coexisting with normal fetus, pregnant 35.3 weeks who presented anemia grade II.

**Diagnosis:**

The initial diagnosis of the mole was by ultrasound.

**Interventions:**

KERR-type cesarean section and bilateral tubal occlusion. The newborn was morphologically normal, and she did not require intervention or treatment.

**Outcomes:**

The newborn was feminine, morphologically normal, weighing 2380 g and 47 cm, APGAR score 8 to 9, delivered prematurely, and there was a large placental plate. The blood loss on surgery was estimated at 1000 mL. Histopathology report of an incomplete hydatidiform mole, negative for malignancy. Histopathology diagnostic was confirmed by immunohistochemistry staining for p57KIP2.

**Lessons:**

Although the incidence of this pregnancy is very rare, early recognition, diagnosis and divulge of the cases of medical community is very important for patient care.

## 1. Introduction

A pregnancy with hydatidiform mole (HM) can be classified into complete or incomplete mole pregnancy, the majority cases are classified how complete HM, and the report of incidence of twin pregnancy with complete mole is 1 in 10,000 to 1 in 100,000 pregnancies.^[1]^ On the other hand, twin pregnancies with incomplete HM, also known as partial mole, coexisting with a live fetus are rarely reported, since fetuses identified with partial mole are generally triploid and tend to miscarriage at first trimester.^[2]^ HM with live fetus is associated with the a risk of a wide variety to maternal and fetal complications from hyperemesis to lethal risk of gestational trophoblastic neoplasia.^[3]^ An early diagnosis, if is possible, of this condition by ultrasound is very important for clinical management and help to patient and her partner to choose a decision whether to terminate pregnancy or continue with close feto-maternal monitoring. The incidence of a normal live fetus and a partial molar such as the case we describe is extremely rare, and it is the first report case in our state.

## 2. Case report

**Figure 1.**
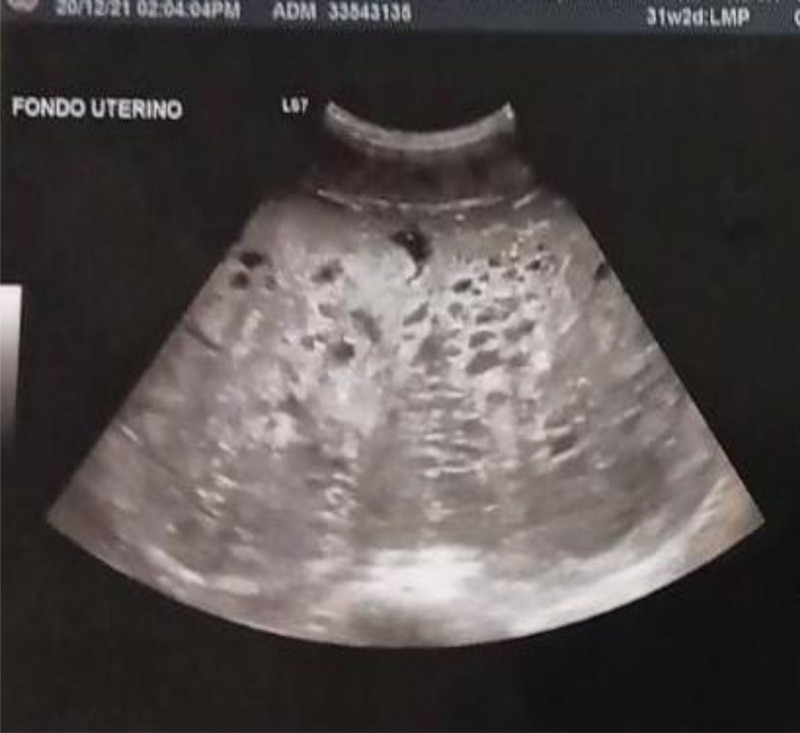
Complex intrauterine mass to rule out partial mole at 31.2 gestational weeks.

We report the case of multiparous of 34 years-old who presented at 35.3 weeks to the emergency labor ward at Women´s Hospital in Culiacan Mexico with a suspected HM and a coexistent live fetus. The pre-referral medical and obstetric history were pregnant woman admitted due to presenting colic type pain, has history of obstetric ultrasound date December 20 2021 which reported pregnancy of 31.2 weeks, pregnancy longitudinal, cephalic, 1 fetus, live, back to the right, 156 beats per minute (BPM) of fetal heart rate, anterior corporal placenta grade II, amniotic fluid index 5 cm^3^, hyperecogenic and complex intrauterine mass is observed to rule out the possibility of incomplete mole (Fig. 1).

Obstetric history; date of last menstrual period may 15,2021, pregnancies; 7, deliveries; 6, menarche 14 years, menstrual cycles; regular 28 × 3, beginning of active sexual life; 18 years, last Papanicolaou diagnostic; 2018 with normal cytology, a total of 3 prenatal checkups, normal ultrasound in the 7th month, micronutrient intake from the first trimester, without history of urinary tract infections or cervicovaginitis, COVID-19 vaccine; 1 dose of Astra Zeneca, negative for SARS-CoV-2 disease, pathological and non-pathological personal history; negatives.

**Figure 2.**
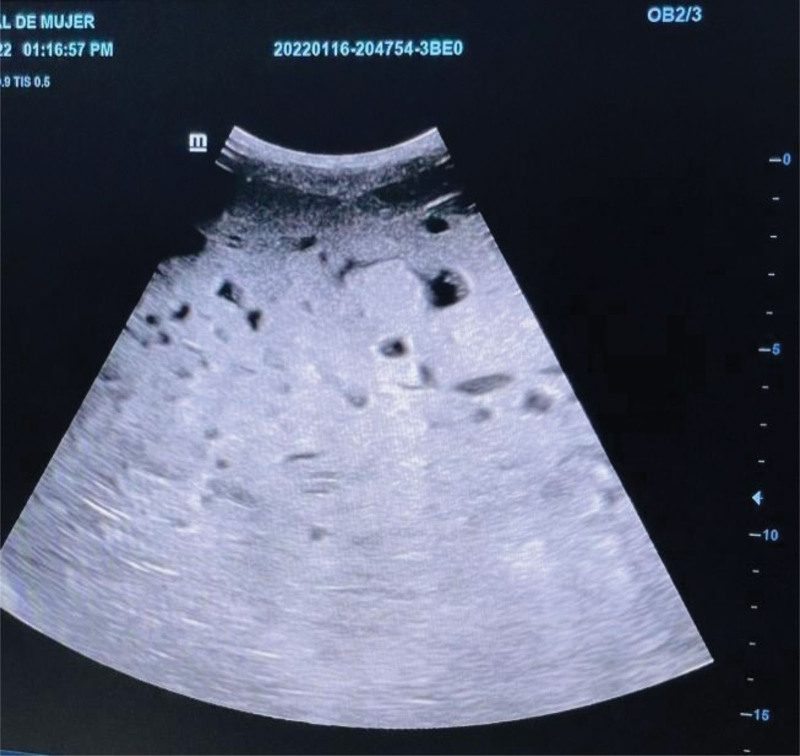
Complex intrauterine mass suggestive of an incomplete mole at 35.3 gestational weeks.

On admission; pregnancy of 35.3 weeks, blood pressure 110/70 mm Hg, temperature 37° C, breathing frequency; 18 per minute, heart rate; 80 BPM. An ultrasound examination detected 1 fetus, live, longitudinal, cephalic, 160 BPM of fetal heart rate, fundus uterus with large placental plate, phometry 34 weeks, 1980 g of estimated fetal weight, a hyperechoic and complex intrauterine mass suggestive of an incomplete mole is observed (Fig. 2). To vaginal touch dilatation of 4 cm, so it is admitted for interruption of pregnancy via abdominal. Her physical exploration was normal vital sings, neurologically integrated, calm, conscious, orientated, with good hydration, slightly pale skin, and integuments, cardiopulmonary without commitment, globous abdomen at the expense of the pregnant uterus, genitals according to age and sex, posterior cervix vaginal feedback, 80% erasure, integral membranes, integral limbs without data of edema, good capillary filling, normal tendon reflexes. Blood tests: red blood cell (RBC) 2.99 million/mm^3^, hemoglobin (Hb) 8.10 g/dL, hematocrit (Ht) 25.70%, white blood cell (WBC) 10,680/mm^3^, platelets (PLT) 232,000/mm^3^, alanine aminotransferase (ALT) 35.1 U/L, lactate dehydrogenase 258 U/L, potassium 4.06 mEq/L, sodium 128 mmol/L, thyroid stimulating hormone 8.320 uUI/mL, free T3 1.97 pg/mL, free T4 1.03 ng/dL, prothrombin time 11 seconds, alkaline phosphatase 323 UI/mL.

The patient did not have human chorionic gonadotropin (β-hCG) test previously and when she was admitted the equipment of laboratory service was in maintenance, we suggested that she undergo β-hCG, amniocentesis and the amniotic fluid karyotype, but the patient refused examinations because of economic reasons. In this regard, it was decided to transfer the patient to cesarean section due to the high risk of presenting uterine atony and potential hysterectomy.

**Figure 3.**
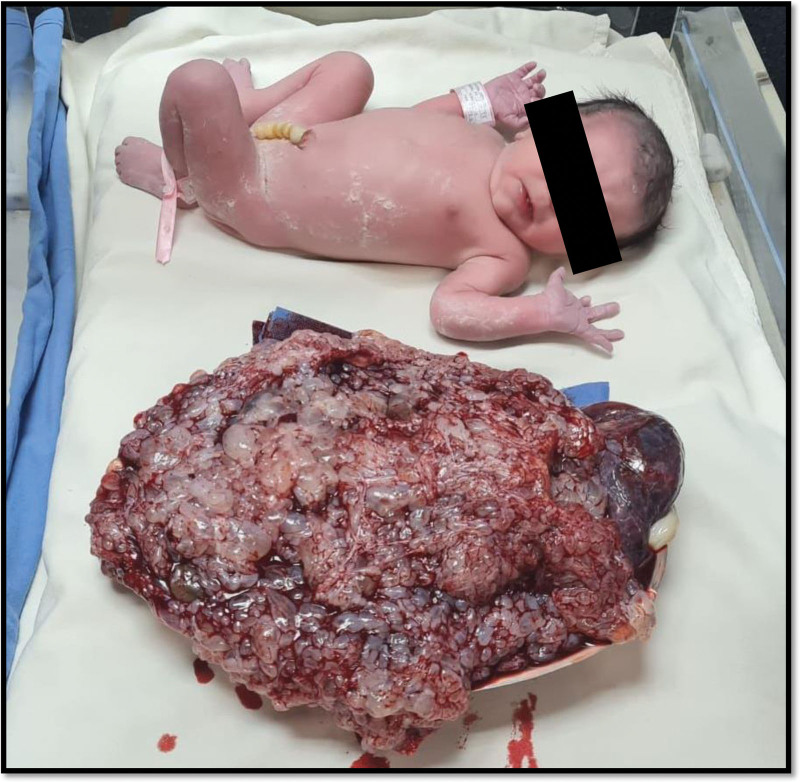
Postoperative findings – newborn (up) and molar tissue (down).

The patient gave her informed written consent to surgical uterine evacuation, uterine curettage, and hysterectomy, if necessary. The procedure was performed under subarachnoid block with ceftriaxone prophylaxis. The blood loss was estimated at 1000 mL. One fetus, live, feminine 2380 g and 47 cm, APGAR score 8 to 9, clear amniotic fluid, normothermal uterine cavity, abundant vesicles are obtained at placental delivery, placenta with incomplete mola weight 3 kg, triple ligature of Tsirulnikov is performed (Fig. 3).

On the first postoperative day, the patient was well with normal vital signs. Blood tests: RBC 3.24 million/mm^3^, Hb 8.70 g/dL, Ht 27.5%, WBC 15,760/mm^3^, PLT 186,000/mm^3^, ALT 29.9 U/L, lactate dehydrogenase 431 U/L. Lactation was not suppressed. The patient was discharged home in good general condition and advised to have β-hCG levels monitored every 7 days.

For 2 postoperative weeks, the patient blood test: RBC 3.77 million/mm^3^, Hb 9.90 g/dL, Ht 32.30%, WBC 7580/mm^3^, PLT 450,000/mm^3^, β-hCG 19.98 IU/mL, ALT 11.9 U/L, creatinine 0.69 mg/dL, potassium 3.95 mEq/L, sodium 138 mmol/L, thyroid stimulating hormone 198 uUI/mL, free T3 2.56 pg/mL, free T4 1.21 ng/dL, alkaline phosphatase 133 UI/mL.

**Figure 4.**
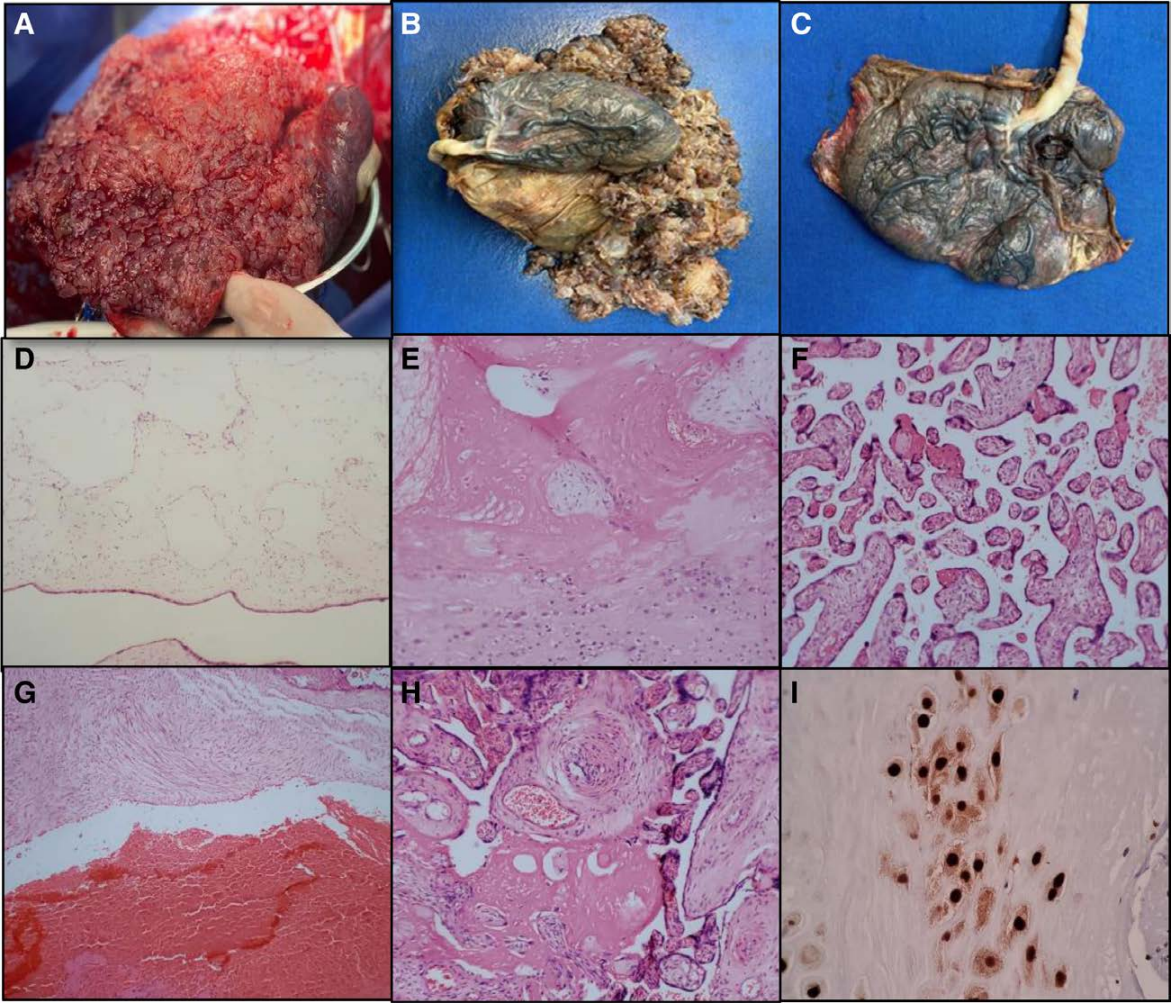
Macroscopic and pathological findings of incomplete mole. (A) Macroscopic image demonstrating scattered multiple cysts. (B) Incomplete molar tissue biopsy of 8.5 × 28 × 30 cm, weighing 2850 g. (C) Placenta of 7 × 22 × 26 cm, weighing 360 g. (D) Amnion with dilated lymphatic vessels, x4 magnification. (E) Decidua and chorionic villi with hyaline degeneration, x4 magnification. (F) Chorionic villi of the third trimester with vascular congestion and focal hyaline degeneration, x10 magnification. (G) Extensive areas of recent hemorrhage, x4 magnification. (H) Chorionic villi of the third trimester with vascular congestion and hyaline degeneration, x40 magnification. Images (D) to (H) with H&E stain. (I) immunohistochemical staining positivity is observed at the nuclear level for p57KIP2, x40 magnification. H&E = hematoxylin and eosin.

Seven weeks later the patient collected the histopathology report of an incomplete hydatidiform mole, negative for malignancy. Additionally, the histopathology diagnostic was confirmed by immunohistochemistry (IHC) staining for p57KIP2 (Fig. 4).

## 3. Discussion

The spectrum of gestational trophoblastic diseases comprises benign forms such as HM to malignant form such as choriocarcinoma, invasive mole, epithelioid trophoblastic tumor and placental site trophoblastic tumor, all of malignant forms are called gestational trophoblastic neoplasia, the most common form of gestational trophoblastic diseases is HM, also known as molar pregnancy.^[4]^

HM occurs as a result of abnormal fertilization and is classified as complete or incomplete based on differences in distinct disease processes with characteristic cytogenetic, histological, and clinical. Complete mole is the most common type and does not contain fetal parts. Most complete moles (80%) occur because of abnormal fertilization of an ovum lacking nuclear deoxyribonucleic acid and have 2 identical paternal chromosome complements derived from duplication of the haploid genome of a single sperm. The remaining 20% occur because of fertilization by a single sperm or via dispermy.^[4,5]^

The incidence of HM varies by geographical region, generally believed that the incidence is higher in developing countries. The incidence is higher in younger than 20 and older than 40 years, also is higher in nulliparous women, in pregnant of low economic status, and in patients whose diets are deficient in protein, folic acid.^[6]^ The report of incidence of twin pregnancy with a complete mole is 1 per 10,000 to 100,000 pregnancies,^[1]^ twin pregnancy with HM and a coexistent live fetus is a very rare condition occurring in 0.005% to 0.001% of all pregnancies,^[7]^ which is possibly due to the high first-trimester miscarriage rate for incomplete moles, as consequences of triploid fetuses.^[2,7]^ In fact, just over 2 hundred cases have been reported in the literature about complete hydatidiform moles with a coexisting fetus in the past 2 decades, and twin pregnancy with a partial mole and viable fetus or a singleton pregnancy with a partial molar placenta are even less common.^[8]^ In this regard, at 2016 only 18 cases of singleton, diploid fetus with incomplete molar pregnancy have been reported.^[9]^

Partial molar pregnancy coexisting with normal live fetus has been divided into 3 types: the most common type class is a twin pregnancy with a normal fetus having a normal placenta and a complete mole; the second type is a twin pregnancy with a normal fetus and placenta and a partial mole; and the third and most uncommon occurrence is a singleton normal fetus with partial molar placenta.^[10]^

For obtain a clinical diagnosis exist techniques such as p57KIP2 immunostaining, fluorescence in situ hybridization, and other advances in diagnostics such as STR genotyping or SNP-based microarray analysis to enhance the diagnosis.^[1]^ In our population these techniques did not carry out because it is a public hospital, and the economic conditions of the patients make these studies even more difficult. Our diagnosis was based on hematoxylin and eosin staining and was confirm using IHC staining for the protein P57kip2 as gold standard, confirmed the diagnosis of an incomplete HM.

The molar pregnancy coexisting with live normal fetus carries a risk of severe maternal and fetal complications. Maternal complications include severe hemorrhage and consequent anemia, severe preeclampsia, thromboembolic and hyperthyroidism disorders.^[11,12]^ Even common are such events as fetal growth retardation, intrauterine death, miscarriage or premature birth.^[13,14]^

## 4. Conclusions

In conclusion, twin pregnancy with incomplete HM and coexisting with normal live fetus is a very unusual condition and the challenge to obstetricians and the consequent successful on these pregnant is early diagnosis. Ultrasound alone is insufficient for diagnosing such as cases but is a good start to identify the initial condition of the pregnant woman. Incomplete HM was visualized previously through ultrasound, this helped when the patient arrives at hospital, confirmed the diagnostic for ultrasound and expediting the protocol and the consequence emergency surgery, the final diagnostics was obtained by IHC.

## Acknowledgements

We are grateful for all the facilities granted by the institute, the team of surgeons, pediatricians and paramedics for the care provided to the mother and newborn.

## Author contributions

**Conceptualization:** Carlos Ernesto Mora-Palazuelos, Luis Garay-Vizcarra.

**Data curation:** Carlos Ernesto Mora-Palazuelos, Paul Gonzalez-Quintero, Erik Rene Lizarraga-Verdugo.

**Formal analysis:** Luis Garay-Vizcarra, Daniel Miranda-Rosas, Eri Peña-Martínez.

**Investigation:** Carlos Ernesto Mora-Palazuelos, Luis Garay-Vizcarra, Paul Gonzalez-Quintero.

**Methodology:** Erik Rene Lizarraga-Verdugo, Saul Armando Beltrán-Ontiveros, Jose Geovanni Romero-Quintana.

**Writing – original draft:** Carlos Ernesto Mora-Palazuelos, Paul Gonzalez-Quintero, Saul Armando Beltrán-Ontiveros, Jose Geovanni Romero-Quintana.

**Writing – review & editing:** Erik Rene Lizarraga-Verdugo, Saul Armando Beltrán-Ontiveros, Jose Geovanni Romero-Quintana.
